# Flowers, Ferns, and Ward Decorations

**Published:** 1886-10-23

**Authors:** 


### Flowers, Ferns, and Ward Decorations.

[Continued from page 47.—Communications for this column should be addressed "Gardener," care of the Editor of The Hospital.]

**Flowers as Symbols**

In distant mythological days different flowers were held sacred to the various deities who supplied exacting humanity with something to worship,—*chacun à son goût*. For instance, the rose was devoted "to Eros and Aphrodite, accounted the emblem of joy and love, and at the same time of prudence"—a well-selected trio, by no means surer to go hand-in-hand then than now! During more modern times the jewels of the seasons have not been unassociated with the great events of the period, since the homely broom gave its name to a line of English kings, and the red and white roses of Lancaster and York were adopted as the outward and visible sign of the rival political opinions of the day.

**The National Use of Flowers.**

The rose, thistle, and shamrock are employed armorially as the national badges of the united realm of Great Britain and Ireland, and very pretty reasons are adduced for their adoption in other years when history was growing. Over the way, since the reign of Clovis, the arms of France have been adorned with fleurs-de-lys, better known as irises or flags. The imperial violet, later on, cannot be said to have in any measure symbolised the race of rulers who assumed it as their badge. America, with its national penchant for things on a large scale, has selected the regal magnolia for its representative flower; the lotos, chosen of India and China, may exceed it in size, if not in sweetness. Japan owns the chrysanthemum as the embodiment of the most exalted honours, the Mikado endowing his worthiest subjects with an order of that name. Owing to the Kaiser's weakness for cornflowers, that bonny blue blossom is rapidly becoming a special favourite in the Fatherland. But ere I bring my list to an end, I must speak of our own delicate primrose, modest and tender-hued, the pet flower of one who loved his country well, who guarded her honour and left it high and spotless—one whose memory is held sacred by an ever-increasing mass of English men and women, who have chosen the primrose to denote their brotherhood, and for a continual remembrance of Benjamin Disraeli's name.

*(To be continued.)*

